# Supplementary material for: Are carbon emissions trading and green financial instruments synergistic? -Comprehensive quantitative research based on content analysis
Source: PLoS One. 2024 Mar 7;19(3):e0298601. doi: 10.1371/journal.pone.0298601 (PMC10919601; doi:10.1371/journal.pone.0298601)
Supplement: S1 Data — (ZIP) [file pone.0298601.s001.zip › Data/地级市Z_F工作报告.zip!/╔╜╢1⁄2/╢1⁄2╙¬╩╨/└┤╫╘╣1⁄2╓┌║┼í╛┬φ┐╦╩2╛▌═°í┐_2008.docx]

各位代表：

现在，我代表市人民政府向大会作政府工作报告，请予审议，并请市政协委员和其他列席会议的同志提出意见。

一、过去五年的工作

过去五年，在省委、省政府和市委的正确领导下，全市上下以邓小平理论和“三个代表”重要思想为指导，以科学发展观统领全局，开拓进取，扎实工作，圆满完成了市五届人大一次会议确定的目标任务，经济社会发展取得了显著成就。

综合经济实力大幅提升。主要经济指标比2002年实现翻番。预计2007年全市生产总值1650亿元，增长1.2倍；境内财政总收入339.7亿元，增长1.9倍，其中地方财政收入60.1亿元，增长2.2倍；社会消费品零售总额203亿元，增长1.1倍。经济结构进一步调整。企业规模不断壮大，年销售收入过10亿元的工业企业集团达到35家。规模以上工业企业销售收入3180亿元、利税815亿元，分别增长4.6倍和3.6倍。服务业较快发展，增加值年均增长18.3%。金融机构存款余额857.5亿元，贷款余额595亿元，分别增长1.2倍和1.8倍。县域经济活力增强。“十五”计划胜利完成，“十一五”规划进展顺利。

社会主义新农村建设扎实推进。农业综合生产能力进一步提高，优势产业和龙头企业不断壮大。培育黄河口农产品品牌取得明显成效，10大特优农产品全部完成商标注册。农业和农村基础设施得到改善，农村基本实现“五通”。农村环境综合整治取得较大成效。“两进”工程顺利实施，初步构建起农村现代商品流通服务体系。农民科技文化素质进一步提高。小康文明村创建活动扎实开展，小康文明村达到438个。

改革开放取得重要进展。农村综合改革逐步深入，农业税、农业特产税全部取消，支农惠农政策得到落实。企业改革继续深化。财税体制改革力度加大，税收和非税收入征管机制进一步健全，财政管理制度不断完善。投融资体制改革实现突破，政府投资管理更加规范。国有资产管理体制、土地管理、金融、价格及社会领域改革取得新成效。非公有制经济活力增强。市场经济秩序明显改善。招商引资水平逐步提高，累计实际利用外资7.9亿美元。对外贸易不断扩大，2007年实现进出口总值24.5亿美元，其中出口13.5亿美元，分别增长3.7倍和3.6倍。经济开发区较快发展。境外投资、对外承包工程和劳务合作迈出新步伐。第三届黄河国际论坛暨首届黄河口旅博会成功举办。

环境建设得到加强。五年新建、改造等级公路5400公里，建成东营黄河公路大桥，威乌高速公路利津段建成通车。水利、电力、通信等基础设施进一步完善。黄河口治理和保护取得明显成效。建成城东和垦东防潮大堤。“数字化东营”建设扎实推进。城市规划建设管理水平不断提高，中心城建成区面积达到75平方公里。启动“黄河水城”建设，广利河综合治理取得重大进展，实现了清水进城的目标。县城和小城镇快速发展，全市城镇化率达到57.4%。生态建设和环境保护取得明显成效，被命名为国家环境保护模范城市。节能减排力度进一步加大。“三网”绿化工程全面展开。

黄河三角洲开发前期工作顺利推进。东营港引桥和2个3万吨级散杂货泊位主体工程基本完成，液体化工品泊位使用港口岸线获得国家发改委、交通部批准。东营机场改扩建工程已经立项开工，正在争取国家民航部门资金支持。一批国内外大企业参与黄河三角洲开发的势头良好，与中海油、正大集团等世界500强企业达成了产业合作意向。市场化运作取得明显成效。与国家开发银行合作顺利推进，东营港对外招商进展良好，黄河国际论坛中心和文化艺术中心投资主体已经确定。资源型城市经济转型试点、生态用水指标、土地综合利用等政策争取工作取得实质性进展。

社会建设富有成效。各级各类教育快速发展，基本普及了学前三年教育和高中段教育，办学条件明显改善，免费义务教育全面实现。油田中小学校顺利移交。基本医疗服务不断健全，公共卫生体系初步形成，公共卫生安全得到保障，人民健康水平不断提高。科技事业较快发展，被命名为全国科技进步先进城市。文化事业取得新的发展，全社会文明程度进一步提高，被授予创建全国文明城市工作先进城市。全民健身运动和竞技体育蓬勃开展。人口和计划生育工作水平不断提高。国防教育、人民防空、民兵预备役和双拥工作进一步加强，外事侨务、民族宗教、对台、档案、史志、无线电管理等工作取得新成绩，妇女儿童、老年人、残疾人权益得到保障，新闻出版、广播电视、红十字会、地名、气象、地震、防灾减灾等各项社会事业取得新进步。基层政权建设得到加强，依法治市扎实推进。“平安东营”建设不断深入，信访工作富有成效，社会治安和油区治安综合治理力度加大，打黑除恶专项斗争深入开展，安全生产形势持续稳定。

人民生活显著改善。城乡居民收入较大增加，城镇居民人均可支配收入由9144元增加到18500元，农民人均纯收入由3166元增加到5810元。就业和再就业工作切实加强，五年新增城镇就业18.3万人，城镇登记失业率一直控制在2%以内。社会保障体系不断完善，实施“五保五救助”取得明显成效，城乡低保达到全省较高水平，建成了一批经济适用房、廉租房和农村特困群众解困房，残疾人救助水平不断提高，困难群众和弱势群体基本生活得到保障。高度重视解决黄河南展区问题，房台扩建、供水工程进展顺利。实施“突破利津”战略的力度不断加大。居民消费结构优化，生活水平进一步提高。

政府自身建设水平有新提高。行政审批制度改革不断深化，精简审批事项，实行一站式服务。政府职能加快转变，促进政府管理创新，效能建设取得成效。依法行政水平进一步提高，行政行为更加规范。决策目标、执行责任、考核监督“三个体系”不断完善。认真贯彻公务员法，公务员队伍建设得到加强。廉政建设和反腐败工作不断深化。

在看到成绩的同时，也要清醒认识到，我们的工作与人民的期待还有不小差距，前进中还面临不少困难和问题，突出的是：经济结构不合理，三次产业中二产过重、三产过轻，工业内部传统产业过重、高新技术产业和先进制造业过轻。发展环境亟待改善，对外交通建设明显滞后。化工企业围城问题比较突出，节能减排压力增大。一些民生问题还没有很好解决，部分群众生活比较困难，影响社会和谐稳定的矛盾和问题仍比较突出。个别部门推诿扯皮、效率低下的问题比较严重，以权谋私问题仍然存在。干部队伍的思想境界、思维方式、工作能力同新形势新任务不够适应。我们要高度重视这些问题，认真加以解决。

总起来说，这五年，是发展明显加快，质量效益不断提高的五年；是民生问题得到改善，人民群众得到更多实惠的五年；是城乡面貌发生深刻变化，社会事业全面进步的五年。

五年来的成就，是省委、省政府和市委正确领导的结果，是市人大及其常委会监督支持、人民政协和各民主党派参政议政的结果，是全市上下、油地军校、社会各界共同奋斗的结果。在此，我代表市人民政府向全市人民和社会各界，向所有为东营建设和发展作出贡献的同志们、朋友们，表示衷心的感谢！

二、新时期的总目标和总战略

东营已经走过了二十四年的光辉历程。二十四年来，东营大地发生了翻天覆地的巨大变化。在一片荒凉贫瘠的盐碱地上，建成了一座新兴的石油城市。全市综合经济实力不断增强，基础设施不断完善，城镇化进程不断加快，城乡面貌发生深刻变化，人民生活水平不断提高，城镇居民收入位居全省前列。

经过二十四年的发展，东营已经站在了一个新的历史起点上，进入了一个新的历史时期。这一时期，是东营由新兴石油城市向现代化黄河三角洲中心城市转变的重要时期。根据党的十七大、省九次党代会和市四次党代会精神，东营新时期经济社会发展的总目标和总战略已经确定。总目标是，到2020年，全面建成小康社会，率先基本实现现代化，建成全国最具活力的生态文明示范区。到那时，东营将成为经济发达城市，综合实力位居全省前列，全市生产总值达到5000亿元，人均生产总值比2000年翻三番，形成以高新技术产业、先进制造业和现代服务业为主体的现代产业体系；交通网络发达，成为区域性交通枢纽，开放程度和国际化水平明显提高；基本实现城乡一体化，生态环境优美，人与自然和谐相处；充分彰显水城特色和黄河口文化内涵，社会事业全面进步，文明程度明显提高。成为宜居城市、和谐城市，人民群众安居乐业。

实现这个总目标，必须认真贯彻落实市委四届三次全委会作出的《决定》，加快实施黄河三角洲开发总战略。实施这个总战略，关系到东营在全省和环渤海地区战略地位的确立，关系到东营的前途和未来。实施这个总战略，必须抓住机遇，迎接挑战。我国经济发展重心加快北移，国家把发展黄河三角洲高效生态经济列入“十一五”规划，省委、省政府作出了加快黄河三角洲开发的战略部署，给我们带来了重大历史机遇。同时，我们面临着资源型城市经济转型和区域间竞争日趋激烈的双重压力。我们唯有乘势而上，迎接挑战，快速崛起，别无选择。实施这个总战略，必须着眼长远，精心谋划。从现在起到2020年，黄河三角洲开发建设分三步走，最关键的是走好第一步，打好基础，快速起步，拉开大规模开发建设的格局，形成快速崛起之势。统筹区域发展，完善规划体系，坚持一体化开发，重点突破“四大主体产业区”和“黄河水城”，以此牵动开发建设。实施这个总战略，必须借助外力，激发内力，凝聚强大合力。

各位代表，同志们！振兴黄河三角洲，是东营人民梦寐以求的愿望，是一项具有重大历史意义的宏伟事业，是历史赋予我们这一代人的光荣使命。要完成这一崇高的历史使命，全市上下必须大力发扬三种精神。一是艰苦奋斗的精神。东营的发展史，就是一部白手起家、百折不挠、历尽艰辛、苦干创业的奋斗史。没有艰苦奋斗，就没有东营的今天。艰苦奋斗是我们的传家宝。二是团结包容的精神。东营的发展史，就是一部油地军校团结合作、四面八方相互包容，共建共兴、共同奋斗的历史。合则强大，和则成功。团结包容是我们的力量源泉。三是勇于探索的精神。东营的发展史，就是一部不断探索、不断开拓、一步一步把事业推向前进的历史。探索才能进取，僵化必然倒退。解放思想、探索进取是我们不断前进的法宝。这三种精神，是东营建市以来形成的宝贵精神财富，是我们克服困难、奋勇前进，不断从胜利走向胜利的强大动力。发扬这三种精神，开拓进取，扎实苦干，我们就一定能开创黄河三角洲光辉灿烂的明天！

三、今后五年的

目标任务和总体要求

今后五年，是加快推进黄河三角洲开发建设的关键时期。我们要全面贯彻党的十七大精神，以邓小平理论和“三个代表”重要思想为指导，深入贯彻落实科学发展观，加快工作指导和经济发展方式转变，坚持走生态文明之路，全力推进黄河三角洲开发建设，推动科学发展、和谐发展、率先发展，夺取全面建设小康社会的新胜利。

今后五年的目标任务是：

——结构调整取得较大进展，经济又好又快发展。全市生产总值年均增长11%以上，达到2800亿元；地方财政收入年均增长15%以上，达到120亿元。初步构建起现代产业体系。工业传统产业整合提升取得显著成效，石化产业整合基本完成，临港产业区30平方公里的化工园区基本建成；高新技术产业和先进制造业快速发展，东营经济开发区软件产业、电子信息产业形成较大规模，打造成高端产业聚集高地，全市高新技术产业产值占规模以上工业总产值的比重达到30%以上。服务业比重达到30%以上，在黄河入海口建成要素完善、独具特色、具有影响力的4a级生态旅游度假区。自主创新能力明显增强，科技进步对经济增长的贡献率达到60%以上。“四大主体产业区”成为新的经济增长极，县域经济更加壮大，区域经济实现协调互动发展。

——社会主义新农村建设取得明显进展，乡村面貌发生更大变化。现代农业加快发展，形成以渔业、畜牧、蔬菜、林果、粮棉为主导产业，标准化、产业化、国际化经营的新格局，农产品加工增值率达到75%以上，基本构建起现代农业的产业体系、生态体系、装备体系、质量安全体系和机制保障体系。农村生产生活条件明显改善，农民素质普遍提高，村风更加文明。

——发展环境显著改善，吸引力和竞争力快速提升。东营港吞吐能力达到3000万吨以上，广利港改造基本完成，德龙烟铁路、黄大铁路全线开通，威乌高速和环渤海高等级公路建成通车，东营机场改造全面完成，建成区域性国际支线机场，构建起高效便捷的立体大交通体系。“黄河水城”全面建成，打造具有文化内涵和东营特色的城市景观，城市功能更加完善。城镇建设进一步加强，城镇化率达到65%以上。服务环境更加优良，成为最适宜创业发展的城市。

——改革开放迈出新的步伐，发展活力增强。各个领域的改革全面推进，体制机制创新取得突破性进展，市场体系进一步健全。对外合作与交流明显加强，黄河国际论坛成为具有影响力的对外交流合作平台，产业合作取得重大突破，更多的世界500强企业落户东营。外贸出口总值年均增长15%，达到27亿美元以上；五年累计吸收外商直接投资12亿美元以上。区域间合作更加紧密。

——文化建设明显加强，市民文明素质全面提高。社会主义核心价值体系深入人心，精神文明建设扎实推进。黄河口文化不断弘扬和发展，成为具有强大凝聚力和影响力的文化品牌。黄河国际论坛中心、文化艺术中心等一批标志性文化设施建成启用，汇聚沿黄文化风情的黄河文化博览馆开工建设。文化事业和文化产业取得新的发展，覆盖全社会的公共文化服务体系基本建立，群众文化生活更加丰富。

——社会建设进一步加强，人民生活全面改善。教育结构与学校布局进一步合理，在巩固完善义务教育的基础上，实现包括学前三年教育在内的12年免费教育。建立起更加完备的医疗卫生保障体系，使人人享有基本医疗卫生服务。年均人口自然增长率控制在7‰以内。社会就业更加充分，每年新增城镇就业3.3万人以上，农村劳动力转移3万人以上，城镇登记失业率控制在2%以内。覆盖城乡居民的社会保障体系更加完善，人人享有基本生活保障。社会主义民主进一步扩大，人民权益更好保障。社会管理体系更加健全，社会更加和谐稳定。城乡居民收入不断增加，生活质量明显改善。城镇居民人均可支配收入年均增长8%，达到27000元以上；农民人均纯收入年均增长9%，达到9000元以上。

——生态文明建设取得明显成效，基本形成节约能源资源和保护生态环境的产业结构、增长方式、消费模式。循环经济进一步发展，能源资源利用效率有较大提高，污染治理取得显著成效，生态环境质量明显改善，让人民群众喝上干净的水、呼吸到清新的空气、吃上放心的食品。黄河口湿地生态实现良性维持。生态文明观念在全社会牢固树立，可持续发展能力增强。万元生产总值能耗和主要污染物排放总量在完成“十一五”目标的基础上进一步下降。“三网”绿化工程基本完成，五年新增造林面积162万亩，林木覆盖率达到25%。2010年，基本建成生态市。

完成上述目标任务，必须以科学发展观为统领，加快工作指导转变，着力提高发展的质量和效益，实现又好又快发展。必须集中力量，着力突破结构、环境、民生三大问题，增创发展新优势。必须充分调动全市上下、油地军校、方方面面的积极性，万众一心，携手共进，为实现我们的目标而努力奋斗。

四、促进经济结构优化升级

坚持把调整经济结构作为主线，加快构筑现代产业体系，切实转变经济发展方式，不断提高经济整体素质和竞争力。着力取得五个“重要进展”：

(一)在构筑现代产业体系上取得重要进展。关键是加快发展高新技术产业和先进制造业，整合提升传统产业，大力发展现代服务业，不断提升产业层次。一是加快发展高新技术产业和先进制造业。重点发展电子信息、新材料、生物医药、石油机械装备等产业。以东营经济开发区为龙头，科学规划，高点起步，集中布局。通过产业招商和现有企业转型，新上一批带动力强的大项目、好项目，培育新的支柱产业，以增量优化带动存量调整。二是整合提升石化、轮胎、造纸、纺织等传统产业。引进战略投资者，扶持骨干企业加快发展，打造支撑力、辐射力强的产业龙头，鼓励中小企业与大企业开展协作配套，延长产业链条，培育产业集群。走新型工业化道路，推动信息化与工业化融合，用信息化和先进技术改造提升传统产业，实施品牌战略，发展高端产品，提高竞争力。科学规划产业布局，促进产业聚集发展，特别是引导化工产业向临港产业区聚集。鼓励和支持企业加快制度创新、技术创新、管理创新，加强企业家队伍建设，推动企业创新发展。三是大力发展现代服务业。这是推进黄河三角洲开发建设的紧迫任务，是解决结构、环境、民生问题的重要出路，是增强地方财力的有效途径，必须放在战略位置来抓，努力提高服务业比重和水平。重点发展生态旅游业、现代物流业和服务外包业。着力发展黄河口生态旅游，带动发展“黄河水城”旅游、滨海滨河旅游、黄河文化旅游，全力打造知名的生态旅游景区和休闲度假胜地，创建成为中国优秀旅游城市。加快物流园区建设，完善物流设施，构建多层次的物流配送体系，加强区域间的物流经济联系，打造区域性物流中心。加快推进软件园建设，强化人才和政策支持，着力培育一批具有国际资质的服务外包企业，积极承接国际服务外包业转移。同时，改造提升商贸餐饮等传统服务业，积极发展金融保险、文化娱乐、商务服务、社区服务等新兴服务业。制定有力的政策措施，突出构建重点城区、重点园区、重点企业“三大载体”，推动服务业集聚发展，提升档次。

(二)在主体产业区建设上取得重要进展。建设“四大主体产业区”，是黄河三角洲开发建设最大的突破口，是推动东营崛起最重要的支撑和最大的经济增长点，必须作为重中之重，举全市之力，加快开发建设。一是集中突破东营港和临港产业区。加快基础设施和生活服务设施配套建设，尽快提高承载能力，重点推进东营港、化工园区、物流园区、生活商贸服务区等重大项目建设。制定政策措施，推动产业北移，促进资金、项目、人才、技术向临港产业区聚集。二是依托黄河三角洲国家级自然保护区，坚持保护与开发并重，建设生态旅游区。重点推进黄河口游船码头、黄河口休闲度假区、黄河口湿地植物园、黄河文化园、湿地生态科教园等项目建设，打造特色旅游品牌。今年重点抓好东营机场到生态旅游区高等级公路、黄河口游船码头及配套服务设施建设，成立旅游开发公司，搭建运作平台。三是建设生态渔业畜牧区。重点建设正大集团生态渔业基地和畜牧基地，带动和辐射规模化标准化养殖，建成全省重要的渔业生态养殖基地、畜产品生产基地和良种繁育基地。四是立足东营经济开发区，突出发展先进制造业、高新技术产业和高附加值服务业。重点建设生态工业园和软件园，建成黄河三角洲先进制造业和高新技术产业的主要承载区。

(三)在建设创新型城市上取得重要进展。一是着力完善区域科技创新体系。鼓励原始创新，突出集成创新和引进消化吸收再创新。加快建立以市场为导向、企业为主体、产学研相结合的技术创新体系，引导和支持创新要素向企业集聚，促进科技成果向现实生产力转化。支持企业加强研发中心建设，加大研发投入。到2012年省级以上研发机构达到35家以上。围绕我市重点产业和重点领域集中攻关，开发关键技术和共性技术。深化油地校科技合作，推动区域科技资源共享共用，充分利用国内外科技资源。加强科技孵化器建设，提高国家大学科技园、高新技术创业服务中心等科技创业园区的管理服务水平，支持生产力促进中心等科技中介服务机构发展，构建科技创新创业公共服务平台。扎实推进国家知识产权示范城市创建市、国家知识产权试点园区建设，努力打造区域性科技创新中心。二是培养造就高素质的人才队伍。深入实施人才强市战略，制定和实施创新人才培养规划，积极引进和培养高层次人才，注重培训和培养技能型人才。三是营造鼓励创新的环境。深化科技管理体制改革，完善促进创新的政策体系、激励机制、市场环境，使创新人才大量涌现。

(四)在推进生态文明建设上取得重要进展。坚持生态文明的发展方向，走生态文明的建设道路，是东营增创发展新优势的根本出路。要加大节能减排力度，加快发展循环经济，推进经济生态化，不断提高能源资源节约和生态环境保护水平。一是推进节能降耗，实现资源集约化开发利用。强化土地利用规划管理，依法严格保护耕地，提高城镇工矿用地集约化利用水平，依法加大国有未利用土地的管理力度，搞好清理和整顿，改变低效无序的状况。大力推进建筑节能。认真落实固定资产投资项目节能评估和审查制度，严把新上项目准入关。采用新技术、新工艺、新设备改造高耗能产业，加快淘汰落后生产能力。落实差别电价和超耗能加价政策。强化全社会节约意识，形成健康文明、节约资源的消费模式。二是加强污染治理和环境保护。重点加强水、大气、土壤等污染防治，改善城乡人居环境。严格执行环境影响评价和“三同时”制度，严格实施项目环保审批，从源头上控制新污染源的产生。全面落实小流域综合治理规划，对境内河流逐条进行治理。加强工业污染防治，加快燃煤电厂二氧化硫治理，努力解决化工企业围城问题。加快污水、垃圾处理等环境基础设施建设。加大矿区环境治理力度。加大环境监察执法和检测力度，严肃查处环境违法行为。三是加快发展循环经济。规划建设生态工业园区，积极推行清洁生产，集中发展资源循环利用项目，构筑园区循环经济体系。实施循环经济“1320”试点示范工程，加强25个循环经济项目和8个循环经济链条的培育，争创国家级循环经济试点城市。四是大力推进“三网”绿化工程。实施林网、路网、水网合一的“三网”绿化工程，是改善东营生态面貌的历史性工程。要进一步完善规划，加大投入，高标准建设，力争通过5年或更长时间的努力，基本实现农田林网化、路域林荫化、水系风景化，打造黄河绿洲，建设生态家园。

(五)在协调区域经济互动发展上取得重要进展。胜利油田、石油大学、济军生产基地都是推进黄河三角洲开发建设的重要力量，都为东营的经济社会发展作出了重要贡献。我们要继续高举团结发展的旗帜，进一步加强油地军校结合，加快推进区域经济社会一体化。要有效整合区域内资金、技术、人才、土地等各种资源，在城市和基础设施建设、科技创新、大项目建设、社会管理等方面，不断探索拓展结合的新路子，集中力量，共同推动大开发大开放。要统筹县域经济发展，进一步落实促强扶弱措施，继续实施“突破利津”战略，促进县域经济协调发展，不断壮大。要进一步树立大市民观念，积极创造条件，搞好服务，使生活在东营这片土地上的所有职工群众的工作生活条件不断改善，共享发展成果。全市上下、油地军校、方方面面更加紧密地团结起来，共同致力于黄河三角洲开发建设，我们的力量将更加强大，我们共有的家园将建设得更加美好！

五、深入推进

社会主义新农村建设

解决好“三农”问题，事关全面建设小康社会大局。要把发展现代农业、繁荣农村经济放在新农村建设的首位，着力提高农业效益，增加农民收入，改善农村环境。

(一)加快构筑现代农业体系，提升产业化水平。树立大农业观念，用三次产业联动发展的思路发展农业。积极培育五大主导产业，突出发展渔业和畜牧业。到2010年建成70万亩的黄河口大闸蟹等现代渔业生产基地，建成一批标准化畜牧养殖示范基地。积极发展休闲观光等新兴农业，五年内培育休闲观光农业基地20个。重点结合黄河标准化堤防建设和南展区改造，规划建设一批以“亲近黄河农家游”为主要特色的观光农业项目；突出地域特色和农家文化，规划建设一批休闲农业项目。进一步加强黄河口农产品品牌培育，抓好质量认证和商标注册工作，提高农产品竞争力。大力发展农产品加工业，集中力量培植一批带动力强的农业龙头企业，创新产业化经营机制，完善利益联结机制，延长产业化链条，促进农产品转化增值。完善农产品流通体系，加快农产品批发市场建设改造和配送中心建设，积极发展各类流通组织，继续实施“两进”工程，推动产销对接。强化农产品生产、加工、销售环节的监管，加强动植物疫病防控，确保农产品质量安全。提高农业社会化服务水平，扶持农民专业合作经济组织发展，推进科技兴农，加强农民培训，搞好农业机械推广和服务，为现代农业发展提供保障。大力发展农村二三产业，促进农村劳动力转移，拓宽农民增收渠道。

(二)加强农村基础设施建设，改善群众生产生活条件。加快灌区延伸和田间节水改造，实施河道综合治理，健全引、蓄、节、排体系。完善乡村公路网络，加强养护管理，提高乡村公路等级和管理水平。提高农村数据通信水平。加强乡村规划建设管理，逐步实现乡村规划全覆盖。搞好农村示范住宅和特困户安居房建设。进一步完善农村集中供水工程，提高入户率和保障率。实施新农村电气化建设计划，继续发展沼气等农村清洁能源。推进农村垃圾、污水无害化处理。进一步加大扶持力度，加快解决黄河南展区问题。

(三)深化农村综合改革，加大支农惠农力度。坚持农村基本经营制度，稳定和完善土地承包关系，依法自愿有偿流转土地承包经营权。深化农村征地制度改革，维护农民的合法权益。推进农村金融体制改革和创新，加快培育农村金融市场，鼓励更多的信贷资金投向农业和农村。积极开展政策性农业保险试点。加快推进农村林权和水利设施等产权制度改革。探索集体经济有效实现形式。深化农场改革，解决好农场发展和农场职工群众生产生活中的问题。巩固完善强化支农惠农政策，扩大公共财政和公共服务覆盖农村的范围，让广大农民群众更好享受发展成果。

六、加快基础设施与城市建设

完善的基础设施和城市功能是加快推进黄河三角洲开发开放的先决条件。今后五年，必须立足于大开放、大发展，加大投入，加快基础设施和城市建设，使服务辐射功能显著提升，发展环境显著改善。

全力突破基础设施。重点推进“三路两港一场”建设。东营港，加快实施一期扩建工程，在建成2个3万吨级散杂货泊位的基础上，今年建设2个5万吨级液体化工品泊位，同时着手规划建设二、三期工程，尽快建成区域性深水大港。配套建设疏港物流体系，大力拓展腹地，提高吞吐能力。广利港，进一步搞好规划论证，今年适时开工，建设集商港、旅游港和渔港于一体的综合性港口。东营机场，加快改扩建工程进度，机场标准由4c级提高到4d级，增开国内航线，开通国际航线。威乌高速公路东营段今年全线贯通，黄大铁路、德龙烟铁路今年开工建设。同时，今年开工建设环渤海高等级公路，规划建设新火车站。加强水利、能源等其它基础设施建设。完善防灾减灾体系，加快防潮大堤建设改造，形成高标准的闭合防潮体系，构筑起安全屏障。加强黄河口治理和水利设施建设。推进国家级中心渔港建设。加强能源建设，重点推进大唐东营电厂和风电项目建设。完善电网设施。深入推进“数字化东营”建设，提高信息化水平。

进一步加强城镇建设。一是推进“黄河水城”建设，构建“九横十纵”城市水网水系，打造城市品牌，提升城市品位。加快实施广利河综合治理工程，打造沿河服务业经济隆起带，今年重点搞好沿岸绿化、景观建设和下游段综合治理，初现水清、岸绿、景美的靓丽景色，同时搞好老广蒲沟和东营河的治理，彻底解决中心城河道污染问题。加快西城环境整治，搞好居民小区的配套完善和城中村改造，不断完善城市功能。加强城市道路建设，完善城市交通体系，优先发展公共交通。推进城市轨道交通规划建设，逐步形成连接东西城，并延伸至东营机场和生态旅游区的高速轻轨交通体系。加快中心商务区建设，增强城市的商务开放功能。完善供水排水、供暖供气、污水与垃圾处理等公用设施。加快完善城市规划体系，理顺城市管理体制，运用现代管理手段，实现高效能管理。二是规划建设滨海新城。依托东营港和临港产业区，科学定位，高起点规划，有序推进功能区建设，加快人口聚集，建设设施完善、生态良好、功能健全的滨海城区。三是加强县城和小城镇规划建设。坚持规划先行、因地制宜、合理布局，搞好基础设施配套建设，不断提高县城建设水平。加强小城镇建设，搞好城乡环境综合整治。坚持园区和城镇建设一起推进，加快城乡一体化步伐。

七、加快推进以改善民生

为重点的和谐社会建设

坚持把民生问题摆到政府工作特别重要的位置，高度关注民生，不断改善民生，促进社会和谐。

(一)积极扩大就业，普遍增加城乡居民收入。实施积极的就业政策，加强政府引导，完善市场就业机制。重点做好下岗失业和关闭破产企业人员再就业工作，积极帮助城乡“双零”家庭和就业困难人员就业。积极做好高校毕业生就业工作。完善支持自主创业、自谋职业的政策措施，促进以创业带动就业，使更多劳动者成为创业者。健全面向全体劳动者的职业教育培训制度，加强农村富余劳动力转移就业培训。建立统一规范的人力资源市场，形成城乡劳动者平等就业的制度。规范和协调劳动关系，落实农民工政策，依法维护劳动者权益。着力提高低收入者收入，逐步提高最低工资标准，建立企业职工工资正常增长机制和支付保障机制。创造条件让更多群众拥有财产性收入。

(二)加快完善覆盖城乡居民的社会保障体系。积极推行城镇居民基本医疗保险，健全城镇职工基本医疗保险和失业、工伤、生育保险制度。加快建立面向农民工和失地农民的社会保障制度，完善农民养老保险制度、农民医疗保险制度、城乡居民最低生活保障制度。提高统筹层次，加强社会保障基金的征缴、使用和监管。积极发展社会福利和慈善事业，完善弱势群体和困难群众的社会救助体系。做好优抚安置工作。完善住房保障制度，搞好经济适用房和廉租房建设，加快解决城镇低收入家庭住房困难。建立经济发展、物价上涨与提高困难群众生活补贴和保障标准联动机制。做到社会保障体系无缝隙覆盖，城乡低保标准保持全省较高水平，凡是上级有政策规定的都以最快的速度、按最高的标准加以落实，弱势群体和困难群众的基本生活得到有效保障，遭受大病大灾的群众得到及时救助。

(三)加快发展社会事业，促进社会全面进步。优先发展教育，促进教育公平，办好人民满意的教育。优化教育资源配置，促进各级各类教育协调发展。高质量高水平实施义务教育，促进普通高中教育科学发展，大力发展中等职业教育，着力提高高等职业教育质量。重视学前教育，关心特殊教育。更新教育观念，实施素质教育，提高学生综合素质。加大财政对教育投入，规范教育收费，进一步健全学生资助制度，保障经济困难家庭、进城务工人员子女平等接受义务教育。加强教师队伍建设，提高整体素质。鼓励和规范社会力量兴办教育。坚持公共医疗卫生的公益性质，强化政府责任和投入，加快建立基本医疗卫生制度，建设覆盖城乡居民的公共卫生服务体系、医疗服务体系、医疗保障体系，为群众提供安全、有效、方便的医疗卫生服务。完善重大疾病防控体系，提高突发公共卫生事件应急处置能力。加强农村三级卫生服务网络和城市社区卫生服务体系建设，加快乡镇卫生院改革，推进乡村卫生一体化服务管理，扩大社区卫生服务覆盖面。加强医德医风建设，提高医疗服务质量。确保食品药品安全。积极发展体育事业和体育产业。以“庆奥运、迎全运”为重点，广泛开展全民健身运动，提高竞技体育水平。办好东营市第八届运动会。坚持计划生育基本国策，稳定人口低生育水平，提高出生人口素质，统筹解决人口问题。加强国防教育，抓好民兵预备役建设，巩固扩大双拥成果。关心支持妇女儿童、残疾人、老年人工作。进一步做好外事侨务、民族宗教、人民防空、无线电管理、档案、气象、对台、地名、史志、红十字会、防震和防灾减灾等工作。

(四)加强社会管理，维护安定和谐的社会秩序。扩大人民民主，保障人民群众享有更多更切实的民主权利。推进依法治市，深入实施“五五”普法规划和“四五”依法治市规划。建立和完善科学的社会服务和管理体系，重视城乡社区建设，切实解决社区工作有人办事、有钱办事、有地方办事的问题。加强城市出租房屋和流动人口管理。完善社会利益协调和矛盾纠纷调处机制，依法加强信访工作，妥善处理人民内部矛盾。强化安全生产管理和监督，预防重特大安全事故发生。完善突发事件应急管理体制机制。进一步健全社会治安防控体系，加强社会治安和油区治安综合治理，深入开展平安创建活动，改善和加强城乡社区警务工作，深化打黑除恶专项斗争，依法防范和打击违法犯罪活动，保障人民生命财产安全。

八、繁荣发展

具有东营特色的先进文化

促进文化发展繁荣，是增强区域综合竞争力的重要因素，也是人民群众的热切愿望。要突出黄河口文化特色，大力加强文化建设，提高文化软实力，让人民群众共享文化发展成果。

(一)打造黄河口文化品牌。黄河文化、海洋文化、古齐文化、石油文化、生态文化在黄河口交汇，构成了具有鲜明特色的黄河口文化。其主要内涵就是艰苦奋斗、百折不挠、团结包容、探索进取，这是激励全市人民不懈奋斗的精神力量，具有鲜明的时代意义。我们要着力打造黄河口文化品牌，不断增强凝聚力和影响力。深入挖掘和研究黄河口文化，推进创新和发展，不断丰富其内涵。大力弘扬和宣传黄河口文化精神，以此教育人民，鼓舞斗志，凝聚力量。打造黄河口文化传播平台，办好黄河国际论坛和黄河口旅博会，发挥沿黄城市旅游文化合作联盟作用，规划建设黄河文化博览馆，汇聚沿黄文化风情，加强对外文化交流，提高东营在国内外的影响力。

(二)大力建设和谐文化。深入开展社会主义荣辱观教育，大力加强思想道德建设，引导群众自觉履行法定义务、社会责任、家庭责任。加强人文关怀和心理疏导，塑造自尊自信、理性平和、积极向上的社会心态，建立健康和谐的人际关系。加强青少年道德教育，广泛开展城乡志愿服务活动，着力丰富农村、进城务工人员的精神文化生活。加强网络文化建设和管理，营造良好网络环境。广泛开展群众性精神文明创建活动，巩固发展创建文明城市成果，不断提高全社会文明程度。

(三)加快发展文化事业和文化产业。坚持把发展公益性文化事业作为保障人民基本文化权益的重要途径，加大投入力度，加快建设公共文化服务体系。加快黄河国际论坛中心、文化艺术中心等文化场馆建设，建成一批标志性文化设施。加强社区和乡村文化设施建设，着力抓好广播电视户户通、社区和乡镇综合文化站建设、文化信息资源共享、农村电影放映、农家书屋“五大工程”。创作更多更好的精神文化产品。积极发展新闻出版、广播影视、文学艺术事业。大力培育发展文化产业，增强文化竞争力。深化文化体制改革，营造有利于出精品、出人才、出效益的环境。

九、大力推进改革开放

改革开放是加快推进黄河三角洲开发建设的必由之路。要围绕区域创新发展，抓住关键环节和重点领域进行改革和试验，完善体制机制，拓展开放的广度和深度，为区域发展注入强大活力。

推进综合配套改革。深化投融资体制改革，充分发挥政府投资的引导作用，完善市城市资产经营公司等平台的运作机制，扩大市场化运作的领域，提高市场化运作水平。支持金融机构改革，加强与国家开发银行等金融机构的合作，积极吸引股份制银行到我市设立分支机构，探索设立产业发展基金，拓宽融资渠道。探索土地使用制度改革，优化土地利用结构和布局，创新土地开发模式，积极争取国家给予土地综合利用方面的政策支持。积极争取国家把我市列为资源型城市经济转型试点，探索促进接续替代产业发展的机制，增强可持续发展能力。积极创建国家可持续发展示范区。深化财税体制改革，强化税收征管，提高税收和非税收入征管水平，加快建设公共财政体系，推进部门预算、国库集中收付等制度改革，强化预算管理和监督，完善财政转移支付制度，全面提高理财水平。完善市以下财政体制，增强基层政府提供公共服务的能力。加强国有资产管理监督，完善各类国有资产管理体制和制度。稳步推进价格改革。推进公平准入，改善融资条件，破除体制障碍，促进个体、私营经济和中小企业发展。完善市场体系，规范市场秩序，加快社会信用建设。继续深化企业改革，完善现代企业制度。加大社会事业领域改革力度。

扩大对外开放。加大产业招商、专业招商力度，突出抓好“四大主体产业区”招商，着力引进“大、高、外”项目，提高招商引资的质量和水平。加强与跨国公司和国家大型企业的战略合作。充分发挥开发区对外开放的窗口和龙头作用，积极争取东营经济开发区升格为国家级开发区，争取设立出口加工区。加快转变外贸增长方式，调整进出口结构，促进加工贸易转型升级，积极培育出口自主品牌。实施“走出去”战略，创新对外投资和合作方式，支持企业在研发、生产、销售等方面开展国际化经营。加强对外交流，促进与国外友好城市的经济文化合作。加强区域经济合作，按照优势互补、互利共赢的原则，加强与京津唐和长三角、珠三角的对接，重点推进产业对接，加强与山东半岛城市群的融合和济南省会经济圈的对接，加强与周边城市的合作与交流。

十、切实加强政府自身建设

加快推进黄河三角洲开发建设，对政府自身建设提出了新的更高的要求。必须大力推进政府管理创新，提高行政效能，树立崭新的精神面貌，担当起历史重任。

进一步解放思想。开创黄河三角洲开发建设的新局面，必须坚持解放思想，以思想的不断解放推动事业的不断发展。着力转变不适应不符合科学发展观的思想观念，着力解决影响和制约科学发展的突出问题，不断提高驾驭全局的能力、统筹发展的能力、开拓创新的能力、抓好落实的能力。切实克服自我满足、自我封闭，追求安逸、不思进取，畏难发愁、萎靡不振等问题，努力做到以宽广的眼界谋划发展，以一流的工作标准和效率推动发展；努力做到勤奋学习，与时俱进，开拓创新；努力做到奋发有为，拼搏进取，永不懈怠，永不满足；努力做到尊重规律，讲求科学，把勇于探索的精神与科学求实的态度结合起来。

进一步转变政府职能。加快推进政企分开、政资分开、政事分开、政府与市场中介组织分开，转变政府管理经济方式。减少和规范行政审批，进一步完善一站式服务机制。健全政府职责体系，完善公共服务体系，推行电子政务，强化社会管理和公共服务，建设服务型政府。加快行政管理体制改革，形成权责一致、分工合理、决策科学、执行顺畅、监督有力的行政管理体制。加快推进事业单位综合配套改革。推进行政效能建设，完善落实服务承诺制、行政问责制和责任追究制，强化效能监察。

进一步推进依法行政。认真落实《全面推进依法行政实施纲要》，严格依照法定权限和程序行使权力、履行职责，依法管理经济社会事务，依法调整利益关系，依法解决矛盾和问题。全面推行行政执法责任制和依法行政考核制度，加快完善行政执法体制，规范执法行为，做到公正执法、严格执法、文明执法。深入实施行政复议法律法规，促进行政机关依法行政，保障公民、法人和其他组织的合法权益。认真贯彻政府信息公开条例，大力推行政务公开，完善新闻发言人制度，畅通政府网站等公开渠道，健全政府信息公开的发布、监督和评议制度，增强权力运行的透明度，保证群众对政府工作的知情权、参与权、表达权和监督权。进一步健全重大事项集体决策、专家咨询、社会公示与听证、决策评估等制度，实行依法决策、科学决策、民主决策。加强政府诚信建设，增强政府的执行力和公信力。

进一步改进作风。面对前所未有的机遇和挑战，面对艰巨繁重的任务，我们要大兴学习之风，加强调查研究，善于学习新知识、研究新情况、解决新问题、推动新发展。要大兴求真务实之风，牢固树立正确的政绩观，不断创造经得起实践、人民、历史检验的业绩；真抓实干，着力在抓落实、见成效、上水平上下功夫；完善决策目标、执行责任、考核监督工作体系，实行严格的责任制，强化督查考核，确保各项工作高质量高效率落实。要大兴团结配合之风，加强团结，顾全大局，注重整体配合，确保政令畅通，努力营造团结和谐的氛围，充分调动一切积极因素，形成推动发展的强大合力。要大兴勤俭节约之风，大力倡导艰苦奋斗，勤俭办一切事业，反对奢侈浪费。

进一步加强反腐倡廉建设。在坚决惩治腐败的同时，更加注重治本，更加注重预防，更加注重制度建设，完善惩治和预防腐败体系，拓展从源头上防治腐败工作领域。进一步完善工程招投标、经营性土地使用权出让、政府采购和产权交易等制度，加大对重大工程、重点领域、重要环节的监督，加强对领导干部特别是主要领导干部、人财物管理使用、关键岗位的监督，确保权力在阳光下运行。坚决纠正损害群众利益的不正之风，切实解决群众反映强烈的问题。坚决查处违纪违法案件，依法严惩腐败分子，以反腐倡廉的实际行动取信于民！

做好政府工作，离不开人大、政协、社会各界和人民群众的大力支持。各级政府要自觉接受人大及其常委会的监督，认真执行人大及其常委会的决议决定，支持人民政协履行政治协商、民主监督和参政议政职能，认真办理人大代表建议和政协提案。加强与各民主党派、工商联、人民团体和社会各界的联系，广开参政议政渠道。加强与人民群众的密切联系，紧紧依靠人民群众，从人民群众中获取不竭的发展动力！

各位代表、同志们！今年是新一届政府的开局之年，也是加快推进黄河三角洲开发建设的重要一年。综观面临的形势，一方面有利条件很多，特别是经过几年的努力，发展的大框架已经拉开，为今年实现更大突破打下了良好基础；另一方面，我们也面临着许多不利因素和压力。我们要准确把握形势、把握大局，努力做好今年的各项工作，既要确保完成全年的各项指标，又要确保黄河三角洲开发建设扎实推进。今年经济社会发展的主要预期目标是：生产总值增长12%，全社会固定资产投资增长18%，社会消费品零售总额增长15%，外贸出口总值增长16%，吸收外商直接投资增长15%，地方财政收入预算安排增长12.3%、工作目标增长15%，城镇居民人均可支配收入增长10%，农民人均纯收入增长10%，城镇登记失业率控制在2%以内，居民消费价格总水平涨幅控制在4%以内。主要约束性指标是：万元生产总值能耗降低4.5%，二氧化硫排放量削减4%，化学需氧量排放量削减4%，人口自然增长率控制在7.4‰以内。做好今年的工作，我们要把思想认识统一到党的十七大和中央经济工作会议精神上来，认真贯彻落实国家宏观调控政策，坚持稳中求进、好字优先、好中求快，努力实现速度质量效益相协调，投资消费出口相协调，人口资源环境相协调，改革发展稳定相协调，促进经济社会又好又快发展。着力把握好四个问题。一是突出经济结构调整这条主线。坚持“区别对待、有保有压”，调整投资结构，把握好资金投向，提高科技创新能力，促进经济结构优化升级。重点发展高新技术产业、先进制造业、现代服务业和现代农业，加快构建现代产业体系。严格控制高耗能、高排放、产能过剩行业发展。整合提升传统产业，特别是抓好石化产业的整合。二是突出大项目建设这个重点。黄河三角洲开发的主战场是“四区一城”，“四区一城”必须靠大项目支撑。今年，要着力抓好10大重点项目建设；同时，力争在引进大项目特别是世界500强企业上实现新突破。有效整合各类生产要素，举全市之力突破“四区一城”，使之成为现代产业的聚集区，加快黄河三角洲开发进程。三是突出环境建设这个关键。加快推进“三路两港一场”建设，集中力量突破交通瓶颈，尽快打开对外交通大通道。加强环境保护，着力改善生态环境。全面改善生活环境、基础设施配套环境和服务环境，为大开发大开放提供有力保障。四是突出改善和保障民生这个根本。把解决群众最关心、最直接、最现实的利益问题作为工作的着力点，扎扎实实为群众办实事、办好事、解难题、送温暖。对于中央确定的解决民生问题的各项政策，确保及时、全面、足额兑现，解决好关系群众切身利益的实际问题。对于今年确定的农产品和食品药品安全体系建设、城乡居民住房保障、改善黄河南展区居民生产生活条件等10件便民实事，确保按期完成，取信于民。

各位代表、同志们！我们肩负着崇高的历史使命，我们正在向着新的奋斗目标迈进，我们正在谱写着东营人民美好生活的新篇章。让我们更加紧密地团结在以胡锦涛同志为总书记的党中央周围，高举中国特色社会主义伟大旗帜，以邓小平理论和“三个代表”重要思想为指导，在省委、省政府和市委的坚强领导下，万众一心，开拓奋进，加快推进黄河三角洲开发建设，为夺取全面建设小康社会新胜利、建设富裕文明和谐幸福的社会主义新东营而努力奋斗！
